# Impact of the microalga *Dunaliella salina *(Dunal) Teodoresco culture and its β-carotene extract on the development of salt-stressed squash (*Cucurbita pepo* L. cv. Mabrouka)

**DOI:** 10.1007/s12298-022-01176-6

**Published:** 2022-04-20

**Authors:** Magda F. El-Adl, Mohamed A. Deyab, Mai A. Ghazal, Abdelgawad Y. Elsadany

**Affiliations:** 1 Botany and Microbiology Department, Faculty of Science, Damietta University, New Damietta City, 34517 Egypt; 2 Cyanobacteria Research Laboratory, Microbiology Department, Sakha Agricultural Research Station-Soils, Water and Environment Research Institute, Giza, Egypt

**Keywords:** *Cucurbita pepo*, Growth, Photosynthetic pigments, Salt stress, Productivity

## Abstract

Salinity is a major threat to crop production and global food security. Algae and their extracts containing bioactive compounds can enhance the salt tolerance of plants, including the salt-sensitive plants. The current study evaluated the efficacy of *Dunaliella salina* (Dunal) Teodoresco culture and/or its β-carotene extract in improving the salt tolerance of squash *(Cucurbita pepo* L. cv. Mabrouka). Amendment of *C. pepo* with *D. salina* culture and/or its β-carotene extract was more effective in alleviating the impact of moderate salinity imposed by seawater dilution of 2.5 dS m^−1^ than either low (0.55 dS m^−1^) or high (3.5 dS m^−1^) salinity, with a comparable effect to that of salicylic acid (SA). Plants that received a combination of *D. salina* culture and its β-carotene extract showed significantly higher growth (total biomass, fruit productivity) and physiological attributes (photosynthetic pigments, nitrogen (N), phosphorus (P), and potassium (K^+^) contents) than those receiving either amendment alone, reaching up to 80–90% of the SA-treated plants at moderate salinity (2.5 dS m^−1^). The combination could enhance the antioxidant activity of moderately salt-stressed *C. pepo* via increasing carotenoids and phenolics contents, suggesting that this combination could enhance the adaptation of *C. pepo* to the moderate salinity. The present study recommends using the blooms of *D. salina* and its β-carotene that is naturally secreted in situ in natural or synthetic open systems in improving the salt tolerance of *C. pepo* instead of using the expensive synthetic hormones.

**Supplementary Information:**

The online version contains supplementary material available at 10.1007/s12298-022-01176-6.

## Introduction

Salinity is portrayed as a major threat to world food security, where it is the most severe factor limiting plant growth, development and metabolism resulting in various physiological, biochemical, and molecular fluctuations, that may cause death to plants (Sehrawat et al. 2019). Salinity imposes two stresses viz. osmotic stress (first phase) that starts within the first hours following the salt stress and ionic stress (second phase) that is resulting from excessive uptake of Na^+^ and Cl^−^ that hinder different metabolic processes via induction of nutritional imbalance or oxidative damage of different biomolecules (Hanin et al. 2016).

The exposure of plants to salt stress all over the world led to global changes and alarmist projections; thereby, in modern agriculture, scientists are searching for safe solutions to enhance plant resilience to salt stress such as external additives (Hernández-Herrera et al. 2013; El-Katony et al. 2020). Although the chemical additives used to improve  the salt tolerance of plants meet the agricultural crop criteria of high yield and good quality, they are costly and unsafe (Gupta et al. 2013).

Instead, there is a cost-effective, simple, and safe method that is exemplified by the beneficial biological additives of algae and/or their extracts. Application of algal additives can alleviate the negative effects of salt stress on plants and improve performance, and overall plant productivity (Gupta et al. 2013; Tiwari et al. 2017).

By virtue of the algal abundance and their high growth rate in different habitats of various countries and having a treasure trove of untapped natural, biologically active compounds including macro-and micronutrients, and signaling molecules, they can be used as nutraceutical supplements, commercial biofertilizers, and bio-stimulants in agriculture to increase plant growth and yield (Mutale-joan et al. 2020).

The algal powder or extract provides multiple benefits to cultivated plants by their application either as foliar spray or soil drench as an organic, slow-release fertilizer or soil amendment to return nutrients (both macro-elements and carbon) in order to improve the physical and biological properties of soil for cultivating plants (Carillo et al. 2020).

Among algal species, *Dunaliella salina* is one of the richest sources of β-carotene, since it is considered as a sustainable feedstock for the commercial production of natural β-carotene (Rammuni et al. 2019). β*-*carotene is a secondary metabolite and a lipid-soluble orange pigment, exceeding 80% of *D. salina* total carotenoids (Raposo et al. 2015). β*-*carotene exhibits several important biological activities either for human health or for the plant, including quenching free radicals under salt stress conditions and preventing oxidative damage to the plant cell, owing to its vigorous antioxidant properties (Rammuni et al. 2019).

*Cucurbita pepo* L. cv. Mabrouka, a member of the family Cucurbitaceae, is a rich source of soluble fibers, pro-vitamin A, protein…etc. Thus, it plays an important role in the human diet (Vural et al. 2000). So, tackling the problem of the negative impact of salinity on *C. pepo* is very crucial for achieving food security. To the best of our knowledge, no studies had been conducted on using the culture of *D. salina* or its β-carotene extract in alleviating salt stress on *C. pepo*. The hypothesis of this research is the use of *D. salina* culture and/or its β-carotene extract to improve the salt tolerance of *C. pepo* via increasing its antioxidant activity. Therefore, this study aimed at testing which *D. salina culture* and/or its β-carotene extract was the best in alleviating the negative effect of salt stress in *C. pepo* (growth and physiological attributes) compared to salicylic acid-treated plants as a standard stress alleviator?

## Materials and methods

### Production of *D. salina* culture

*Dunaliella salina* (Dunal) Teodoresco was isolated from a hyper-saline lagoon located between 31°7′89″N and 29°46′7.40″E in El-Agamy, Alexandria, Egypt in 2018. A sample of *D. salina* was grown on Johnson solution (JS) (Johnson et al. 1968) contained: NaCl 73 g L^−1^, MgCl_2_.6H_2_O 1.5 g L^−1^, KCl 0.2 g L^−1^, CaCl_2_.2H_2_O 0.2 g L^−1^, KNO_3_ 1 g L^−1^, NaHCO_3_ 0.043 g L^−1^, KH_2_PO_4_ 0.035 g L^−1^, Fe-solution (Na_2_EDTA 189 mg L^−1^, FeCl_3_.6H_2_O 244 mg L^−1^), 10 mL, and then 10 mL of microelements solution: H_3_BO_4_ 61 mg L^−1^, (NH_4_)_6_ Mo_7_O_24_.4H_2_O 38 mg L^−1^, CuSO_4_.5H_2_O 6 mg L^−1^, CoCl_2_.6H_2_O 5.1 mg L^−1^, ZnCl_2_ 4.1 mg L^−1^, MnCl_2_.4H_2_O 4.1 mg L^−1^. *D. salina* culture was exposed to nitrogen deficiency concentration (0.25 g L^−1^ KNO_3_). The medium pH was adjusted at 7.5 ± 0.1 and incubated at 28 ± 2 °C under continuous illumination (50 μmol photons PAR m^−2^ s^−1^) for nine days. Three replicates were used for each treatment. The nitrogen deficiency concentration for the best production of β-carotene from *D. salina* was 0.25 g L^−1^ KNO_3_ (Deyab et al. 2021).

### Maximizing *D. salina* growth in an open system (glass basin)

Ten mL of the N deficiency-treated *D. salina* subculture containing 10^7^–10^8^ cells mL^−1^ was inoculated into 100 mL of autoclaved medium (for 20 min at 121 °C), incubated for nine days, and then used as an inoculum in 1L of the medium that was incubated for nine days and then it was transported to 20-L white polyethylene container containing 20 L media that was incubated for nine days. Thereafter, it was inoculated into a glass basin of 120 × 60 × 60 cm containing 100 L medium and incubated for nine days (Geries and Elsadany 2021).

### Extraction of β-carotene from *D. salina* cells

After the incubation period, an aliquot of *D. salina* cells growing in the glass basin was harvested by centrifugation at 10,000 rpm for 5 min, and then filtered through filter paper (Whatman no. 4), and air-dried. The β-carotene content of dried *D. salina* was 0.65 ± 0.01 mg g^−1^ DW (dry weight). The β-carotene was extracted according to the method described by Suzuki (1996) with some modifications. A known weight (1 kg) of dried *D. salina* powder was washed with ethanol (5 L) two times to remove vegetable oil. β-carotene was extracted with hexane (9 L) from the residue, and the hexane layer was evaporated to obtain dark brown semisolid or solid material. To this material, hexane (1 L) was again added, after which the mixture was stirred at 0 °C for 1 h. The precipitated solid materials were removed by filtration. The filtrate was evaporated to obtain dark brown semisolid. Again, hexane (185 mL) was added to this semisolid, and the mixture was placed at -20 °C overnight. Solid materials containing impurities precipitated out were removed by filtration. The filtrate was subjected to column chromatography to recover β-carotene.

### Separation of β-carotene on silica gel column and confirmation by TLC

The filtrate obtained from the hexane extraction at − 20 °C was directly subjected to silica gel column chromatography (silica gel, 900 mL; column, 5.5 cm × 38 cm; elution solvent, hexane–ethyl acetate (50:1, v/v) to remove impurities. The β-carotene was collected using a mobile phase containing (9:1) hexane to ethyl acetate. The β-carotene-rich fraction was concentrated and treated with ethanol to obtain an orange powder. When the β-carotene-rich fraction eluted from the column was placed in dark for a few hours. TLC was used to confirm the analyzed fraction against standard β-carotene using a chamber containing 70% hexane and 30% acetone. TLC confirmed the carotene-rich fraction is indeed β-carotene (Suzuki et al. 1996).

### Amendments preparation and application techniques for β-carotene (200 ppm)

The extracted β-carotene (1 g) was dissolved in 60 mL of methanol and diluted to 100 mL with acidic water (pH 6) to prepare the stock solutions. To make the β-carotene working solution for the sprinkler; an aliquot (20 mL) of the stock solution was diluted to 1 L of β-carotene solution (200 ppm) with distilled water. The control group was treated with the background methanol solution without β-carotene. A preliminary experiment was conducted to obtain the suitable concentrations of β-carotene where seeds were randomly placed in Petri dishes (15 cm diameter, 15 seeds per dish) containing filter paper moistened with 10 mL of 0, 50, 100, 150, 200, 250, and 300 ppm of β-carotene extracted from *D. salina* at 22 ± 2 °C for 5 days. Based on the results of the seed germination test, the best concentration of β-carotene was 200 ppm.

### Culture of *D. salina*

From the previous open system conducted for maximizing N deficiency-treated *D. salina* growth, 10 mL of ten days-old *D. salina* culture was obtained to amend the cultivated plants. The β-carotene content of N deficiency-treated *D. salina* cells was 1.82 ± 0.02 mg g^−1^ FW.

### Salicylic acid (200 ppm)

The stock solution of salicylic acid (SA) was prepared by dissolving one gram of SA in 100 mL of distilled water. Twenty mL of SA stock solution was diluted to 1 L to use as foliar spraying on the *C. pepo* at the 50^th^ day after the seed sowing and also 40 mL of stock solution was diluted by distilled water to 2 L to use as foliar spraying on the *C. pepo* at the 70^th^ day after the seed sowing.

### Plant material

The experiment was conducted during the period from 10 October 2018 to January 2019. The experimental site was located between 31°0.0′0.0″N and 31°0.0′0.0″E. Seeds of squash (*Cucurbita pepo* L. cv. Mabrouka) were obtained from the Agricultural Research Center, Sakha Research Station, Kafr El-Sheikh Governorate; Egypt. Seeds were screened by handpicking to obtain uniform and healthy seeds to reduce the experimental errors resulting from the heterogeneity of the starting materials. Afterward, seeds were surface-sterilized by soaking in 10% (v/v) Chlorox for 20 min and then washed thoroughly with sterilized distilled water.

### Growth conditions

The trays used for seedling growth were filled with a mixture of peat moss and vermiculite (1:1) that enriched with chemical fertilizers; ammonium sulfate (NH_4_)_2_SO_4_, calcium superphosphate (P_2_O_5_), potassium sulfate (K_2_SO_4_) that were added as 1:1:4 and also fungicides were added as 50 g of a fungicide for each 50 kg of peat moss with 50 mL of Hoagland nutrient solution as a source of trace elements. The seedlings were grown at 27 °C for 15 days in the greenhouse in October of autumn 2018 at Sakha Agricultural Research Station. The seedlings were daily exposed to foliar spraying until they were transplanted in pots.

### Field experimental design

After the appearance of the fourth true leaf, the seedlings were transplanted into plastic pots (25 × 30 cm) filled with 8 kg of a silty-clay soil (pH 7.86; EC 2.25 dS m^−1^) saturated with water. Seawater was filtered by cotton clothes to remove stone and fibers. Seawater was mixed with irrigation water by 1:42 and 1:27 (seawater: irrigation water). The salinity of irrigation water and mixed water was 0.55, 2.5, and 3.5 dS m^−1^, respectively.

Sixty-three pots were divided into three groups according to the salinity treatments; the first group, which accounts for one-third of the total pots (21 pots), was watered by irrigation water (0.55 dS m^−1^). Meanwhile, the second and third groups (21 pots for each group) were irrigated with mixed water with EC 2.5 and 3.5 dS m^−1^, respectively.

The seedlings received 0.3–0.5 g of multi-nutrient fertilizer, containing ammonium sulfate, calcium super-phosphate, potassium sulfate that was added by 2:1:2, respectively, and 50 mL of Hoagland nutrient solution (Smith et al. 1983) as a source of trace elements during the growth period. Plants were grown in a greenhouse at the Sakha Agricultural Research Station with 200 µmol m^−2^ s^−1^ irradiance from sunlight in a 14/10 h light/dark period, with day/night temperature of 22/15 °C and relative humidity of about 60–80%.

### Experimental treatments

The plants were irrigated twice weekly until the complete harvesting of fruits at the last stage of development (or up to 90 days of the seed sowing). The three groups of plants were treated with the following treatments; no amendment, 200 ppm β-carotene, a culture of *D. salina*, 200 ppm β-carotene plus the culture of *D. salina*, 200 ppm SA. All these treatments were applied only two times along the experimental period on 50^th^ and 70^th^ days of the seed sowing. β-carotene and SA were applied as foliar spraying whereas the culture of *D. salina* (10 mL) was added to the soil.

### Harvest and growth measurements

After completely harvesting (three times per week), all fruits of *C. pepo* were collected and weighed to estimate their fresh weights (FW), and then oven-dried at 80 °C for 2 days to estimate their dry weight (DW). Also, the FW, DW and total biomass (root, shoot, and fruit) were estimated, and the fresh and dry weights were estimated as g^−1^ plant^−1^.

The photosynthetic pigments (*Chl a*, *Chl b,* and carotenoids µg g^−1^ FW) of *C. pepo* leaves were determined at the vegetative (after 35 days of the seed sowing), flowering (after 45 days of the seed sowing), and harvesting (after 75 days of the seed sowing) stages. The fresh and dry biomass (root, shoot, and fruit) of plants, as well as the cumulative fresh and dry weight of fruits, were determined after harvesting. Also, shoot N, P, K, and phenolic compounds content of the plant were estimated and expressed as mg g^−1^ DW.

### Estimation of harvest index (HI)

Plant fruits were sampled during the flowering stage. The fresh weight (FW) of fruits was weighed and then dried at 80 °C for 24 h. Harvest index (HI) was estimated as the following equation:-

$${\text{Harvest}}\;{\text{ index }}\;\left( {{\text{HI}}} \right) = \frac{{{\text{Fruits}}\;{ }\left( {{\text{FW}}} \right)}}{{{\text{Total }}\;{\text{above }}\;{\text{ground }}\;{\text{biomass}}\;{ }\left( {{\text{FW}}} \right)}}$$

Total above ground biomass: (shoots and fruits) was reported in gram per plant.

### Estimation of water content (WC%)

After collecting plant leaves under controlled conditions, the leaves were weighed and then dried in an oven at 80 °C for two days and DW was measured. Water content (WC%) was calculated from the following equation. Five replicates were used for treatment.

$${\text{Water }}\;{\text{content }}\;\left( {{\text{WC\% }}} \right) = \frac{{\left( {{\text{FW}} - {\text{DW}}} \right)}}{{{\text{FW}}}} \times 100$$

### Estimation of photosynthetic pigments

The photosynthetic pigments (*Chl a,* *Chl b* and carotenoids) of *C. pepo* leaves, from the three stages, were determined using the spectrophotometric method recommended by Lichtenthaler (1987). A known fresh weight of the plant leaves was homogenized in 85% (v/v) acetone and then kept for 6 h in a refrigerator. After centrifugation for 5 min at 14,000 rpm, the supernatant containing the pigments, was quantitatively diluted to known volume with 85% (v/v) acetone, and then the absorbance was measured using Perkin Elmer UV spectrophotometer at three wavelengths of 452, 644, and 663 nm against 85% (v/v) acetone as blank. The concentrations of *Chl a*, *Chl b*, and carotenoids were calculated using the following equations and the results were expressed as µg g^−1^ FW.

*Chl a* = 10.3 A_663_ − 0.918 A_644_

*Chl b* = 19.7 A_644_ − 3.87 A_663_

Carotenoids = 4.2 A_452_ − (0.0264 *Chl a* + 0.426 *Chl b*).

### Estimation of nitrogen content

Shoot nitrogen content was assessed by using micro-Kjeldahl apparatus according to Page et al. (1982) method. Fifteen mL of 40% NaOH was added carefully to a known volume of the digested samples and the distillation was then started. The liberated ammonia was received in 25 mL of 4% boric acid and Bromo-cresol green-methyl red as an indicator (8 parts of 0.1% Bromo-cresol green in 96% alcohol and 1 part of 0.1% methyl red in 96% alcohol). After distillation, ammonia was titrated against 0.04 N sulfuric acid. A blank, containing all reagents except the sample, was prepared and the quantity of acid used in titration was subtracted from the sample values. Nitrogen was expressed as mg g^−1^ FW.

### Estimation of phosphorus content

Total phosphorus was determined in digested samples of  squash shoots following the protocol described by Snell and Sneu (1967). An aliquot of the digested samples (5 mL) was poured into a 25-mL volumetric flask, and then 5 mL of 2.5% ammonium molybdate solution was slowly added and shaken gently. One mL of 0.5% hydroquinone and one mL of 20% sodium sulphite (Na_2_SO_3_)  were added, and then the mixture was shaken and completed to the final volume with distilled water. The solution was shaken vigorously and then the absorbance was measured at 660 nm after 15 min. Phosphorus was determined from the standard curve using KH_2_PO_4_ and expressed as mg g^−1^ FW.

### Estimation of potassium content

Shoot potassium content was assayed in the digested plant samples using a Jenway PFP7 flame photometer following the protocol described by Jackson (1967). Potassium was expressed as mg g^−1^ FW.

### Determination of phenolic constituents

Phenolic compounds were extracted from dried tissues according to the method adopted by Sauvesty et al. (1992). The dried powdered tissue (0.5 g) was extracted three times with 50% ethanol at 40 °C for 4 h. Each extract was centrifuged for 15 min at 3,000 rpm. The clear supernatants were combined, then reduced under low pressure at room temperature, and made up to 3 ml with distilled H_2_O. This extract contains free and conjugated phenolic compounds. The phenolic glycosides were hydrolyzed with 1 mL of 2 N HCl in a boiling water bath for 1 h to cleave the glycoside linkage to sugar and phenolic aglycone compounds. The mixture was neutralized with 0.1 N NaOH and diluted up to 20 mL with distilled H_2_O.

The Folin–Ciocalteau phenol method (Lowe 1993) was used for the phenolic aglycone determinations. One mL of the phenolic extract was mixed with one mL of 10% Folin–Ciocalteau phenol reagent and one mL of 20% anhydrous sodium carbonate and then diluted up to a known volume with distilled water. The absorbance of the blue color was measured after 30 min at 650 nm. Substraction of phenolic content after and before acid hydrolysis gave the content of phenolic glycosides. The blank sample was prepared by using distilled H_2_O instead of phenolic extract. The phenolics content was obtained from the standard curve of pyrogallol and calculated as µg g^−1^ DW.

### Statistical analysis

The experiment was factorial with two factors and three replications (n = 3) in a completely randomized design (Table S1). Two-way ANOVA with Fisher’s least significant difference (LSD) at a significant level *P* value ≤ 0.05 using Sigma Plot V11.0 and SPSS V 18.0 (Snedecor 1956)) was applied to assess the effect of the main factors; 1) amendments with five levels (no amendment, β-carotene, culture of *D. salina*, β-carotene plus culture of *D. salina*, SA) and 2) salt stress with three levels (0.55, 2.5 and 3.5 dS m^−1^), and their interaction on the growth performance and physiological attributes of *C. pepo*. Mean separation was performed according to Duncan’s multiple range test at *P* value < 0.05.

## Results

### Growth of salt-stressed *C. pepo* in response to amendments

**Fig. 1:**
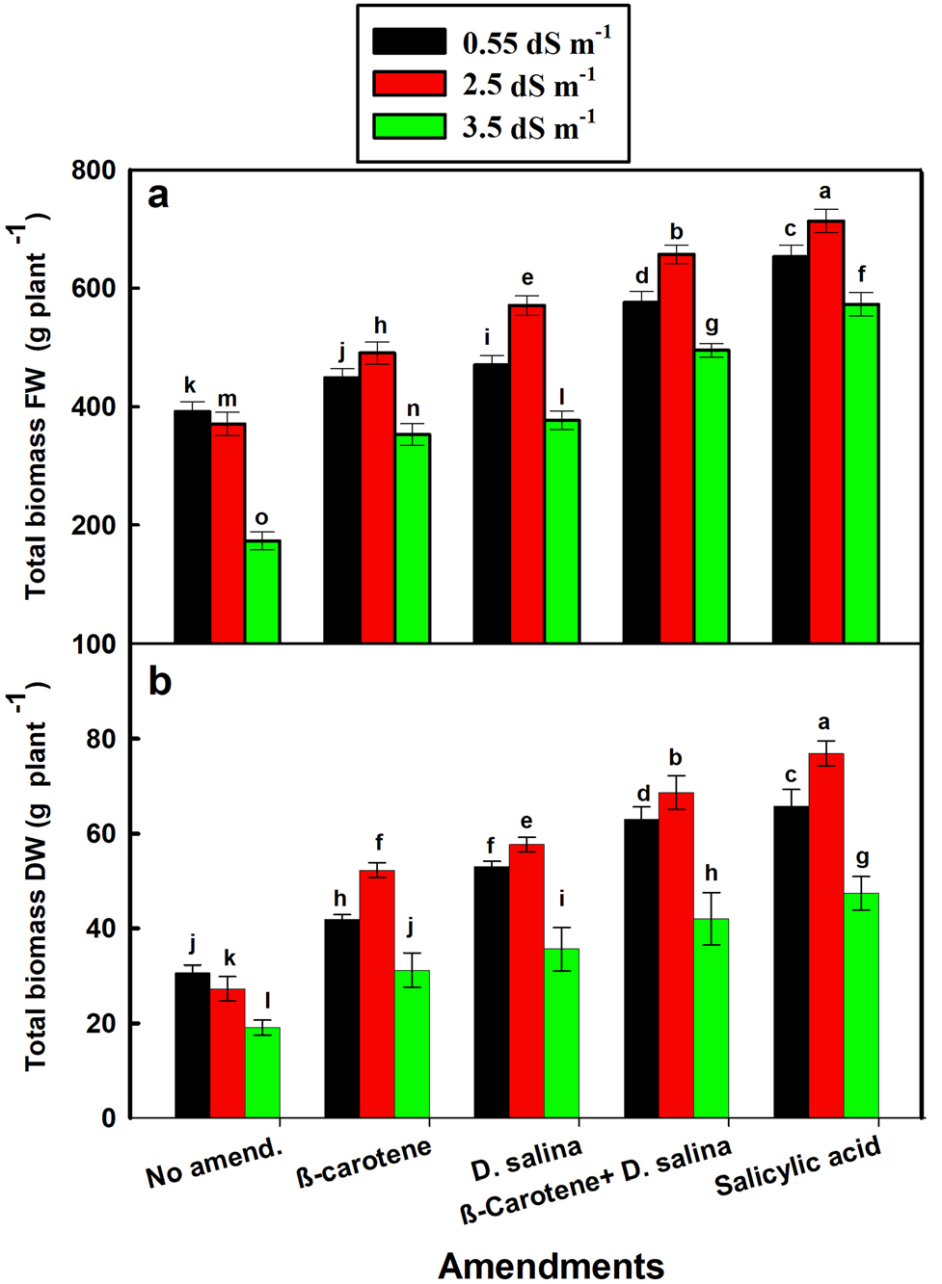
Fresh and dry biomass of *C. pepo* (L.) irrigated with graded dilutions of seawater and amended with different foliar amendments. β-carotene and salicylic acid were applied as foliar spray while *D. salina* culture was added to the soil. **a** Fresh biomass, **b** Dry biomass. Data are mean ± SE. Data labeled with different letters are significantly different at *P* value ≤ 0.05

**Fig. 2:**
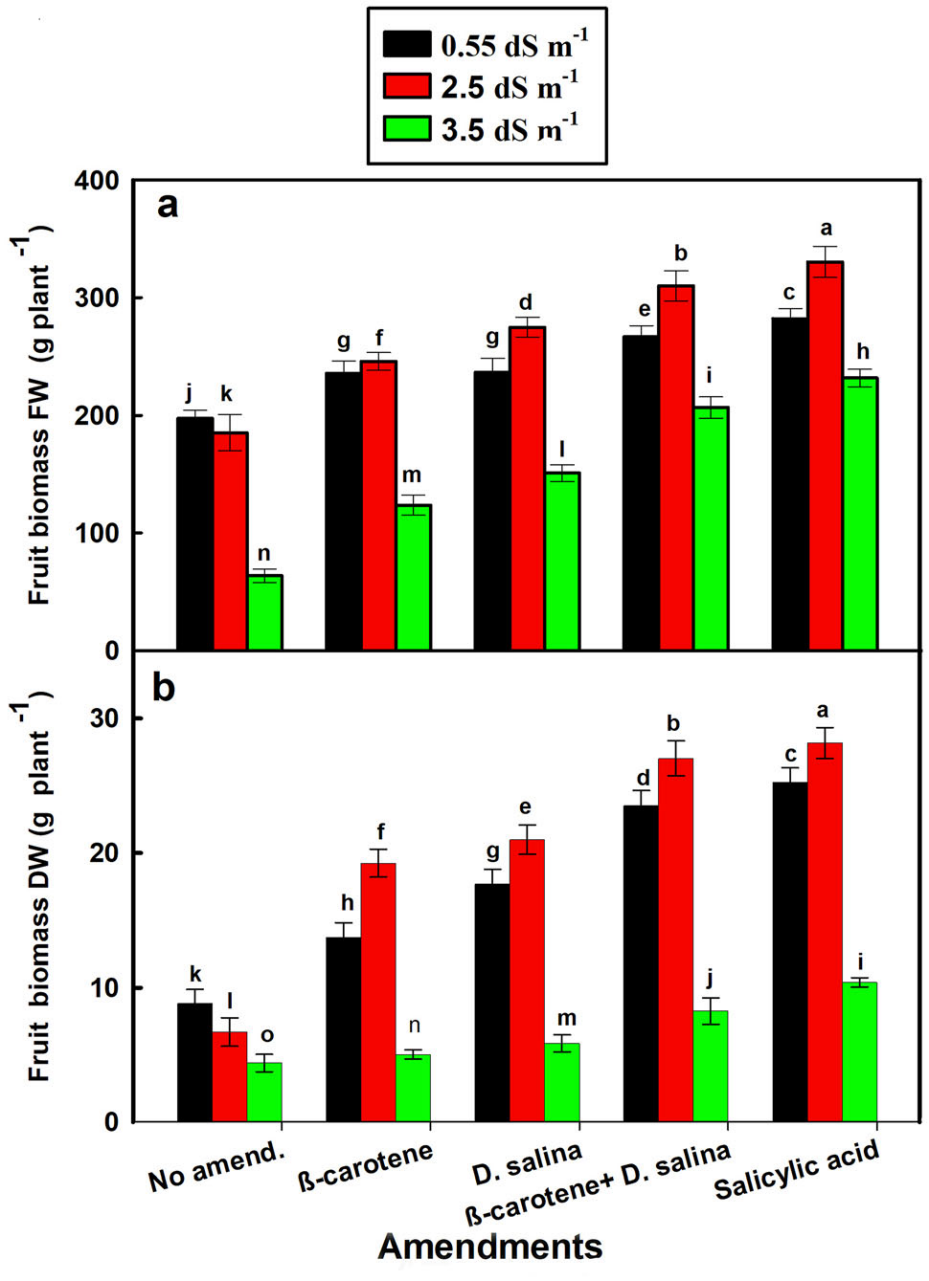
Fresh and dry fruits of *C. pepo* (L.) irrigated with graded dilutions of seawater and amended with different foliar amendments. β-carotene and salicylic acid were applied as foliar spray while *D. salina* culture was added to the soil. **a** Fresh fruits, **b** Dry fruits. Data are mean ± SE. Data labeled with different letters are significantly different at *P* value ≤ 0.05

The response of salt-stressed *C. pepo* growth to amendments varied according to the levels of salt. The total biomass and fruit biomass (FW and DW) of *C. pepo* exhibited a similar pattern, with the differential effect of the combination of *D. salina* culture and its β-carotene extract at the moderate salt level (2.5 dS m^−1^) (Figs. 1a, b, 2a, b). The beneficial effect of the combination of *D. salina* culture and β-carotene was comparable to SA-treatments, since this combination increased the FW and DW of total biomass by 77% and 1.5 folds versus 90% and 1.8 folds for SA, respectively, and increased the FW and DW of fruits by 70% and 3 folds versus 80% and 3.2 folds for SA, respectively, compared to the non-amended plants (Figs. 1a, b, 2a, b).

### Effect of salinity and amendments on photosynthetic pigments

**Fig. 3:**
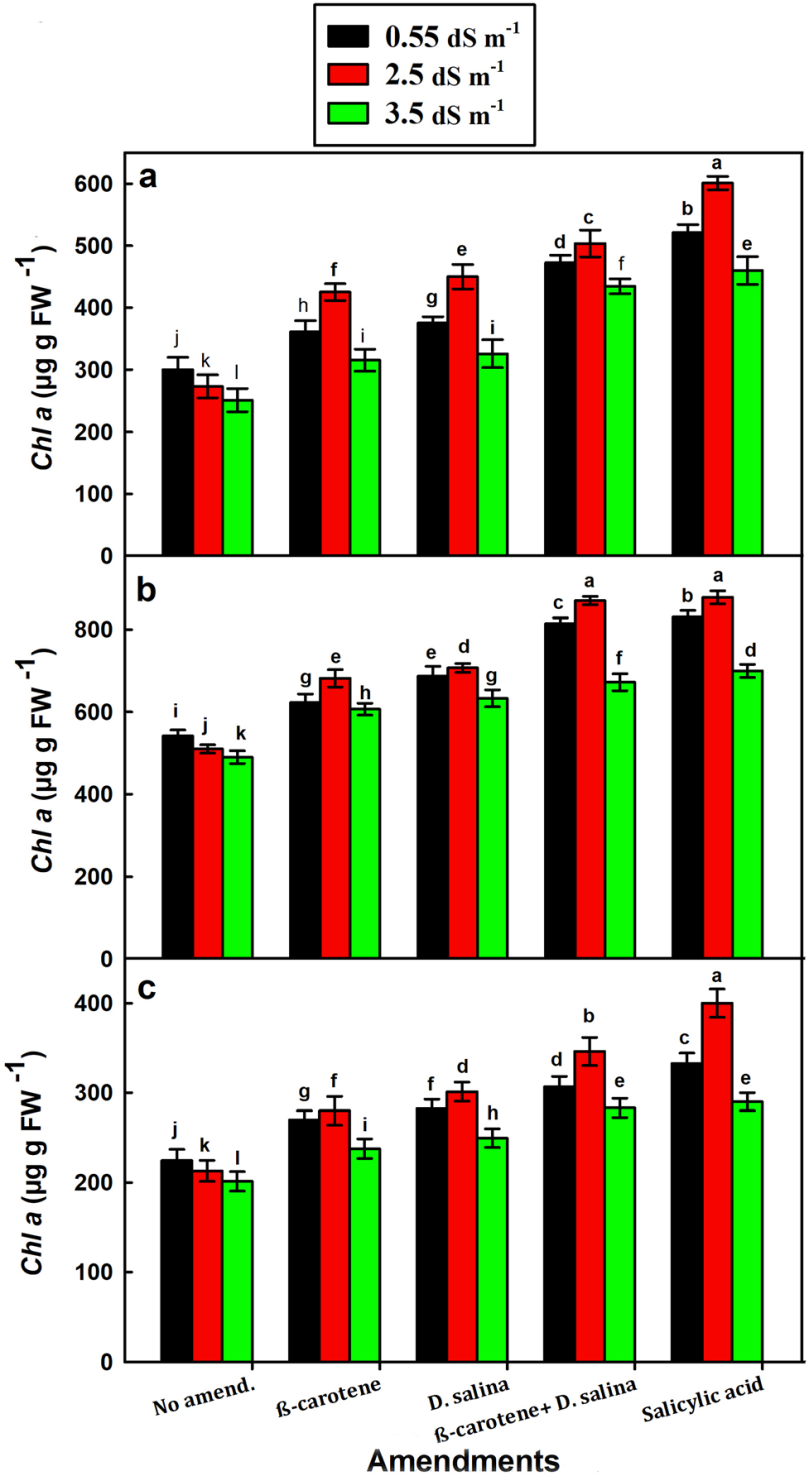
*Chl a* content in *C. pepo* (L.) irrigated with graded dilutions of seawater and amended with different foliar amendments. **a** Vegetative, **b** Flowering, **c** Harvesting stage. β-carotene and salicylic acid were applied as foliar spray while *D. salina* was added to the soil. Data are mean ± SE. Data labeled with different letters are significantly different at *P* value ≤ 0.05

**Fig. 4:**
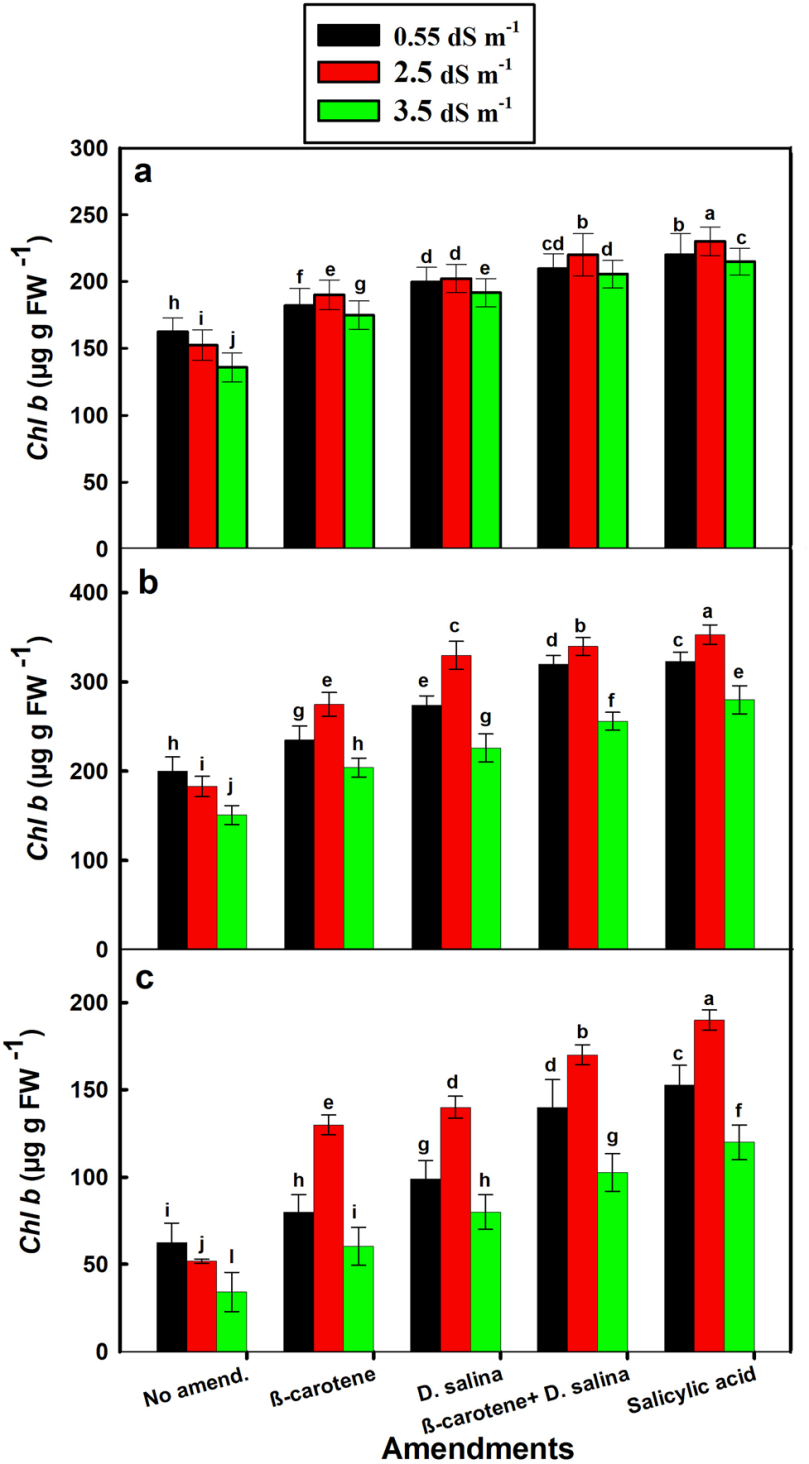
*Chl b* content in leaves of *C. pepo* (L.) irrigated with graded dilutions of seawater and amended with different foliar amendments. **a** Vegetative, **b** Flowering, **c** Harvesting stage. β-carotene and salicylic acid were applied as foliar spray while *D. salina* was added to the soil. Data are mean ± SE. Data labeled with different letters are significantly different at *P* value ≤ 0.05

The flowering stage exhibited significantly higher *Chl a* and *Chl b* content than the vegetative stage, followed by the harvesting stage (*p* < 0.001) at both amended and non-amended plants. *Chl a* and *Chl b* contents in the three stages decreased gradually with increasing salinity from 0.55 to 3.5 dS m^−1^ in the non-amended plants, whereas it increased significantly with increasing salinity in the amended plants, particularly at 2.5 dS m^−1^ (Figs. 3, 4). Within the different amendments, the combination of *D. salina* culture and its β-carotene extract exhibited better beneficial effect than each alone at 2.5 dS m^−1^, where it increased *Chl a* content by 80%, 71%, and 60% versus 1.2 folds, 72% and 9% for SA (Fig. 3a–c), and also it increased *Chl b* content by 44%, 86%, and 2.3 folds versus 51%, 93%, and 2.7 folds for SA in the vegetative, flowering and harvesting stages, respectively, compared to the non-amended plants (Fig. 4a–c).

**Fig. 5:**
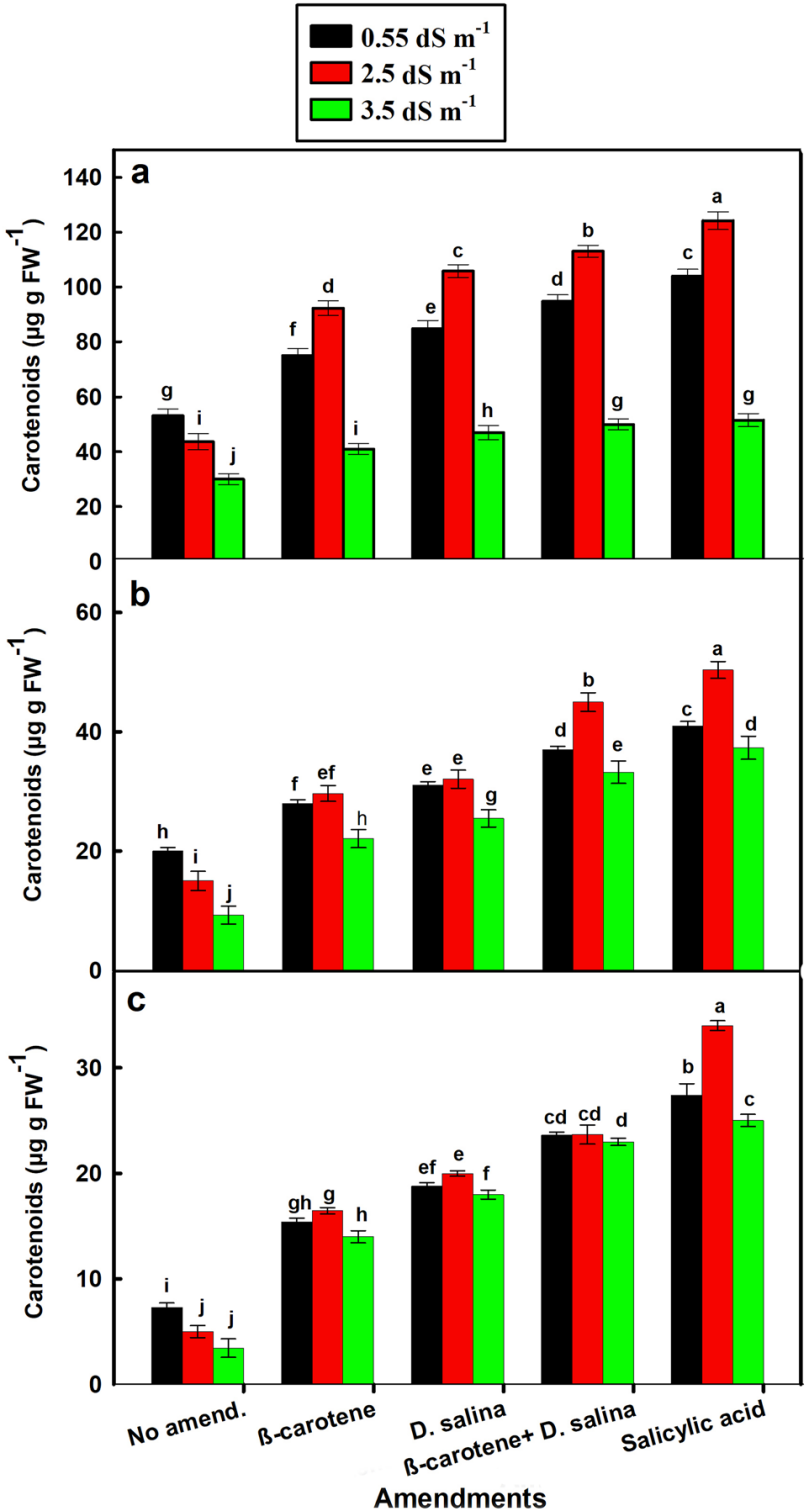
Carotenoids content in leaves of *C. pepo* (L.) irrigated with graded dilutions of seawater and amended with different foliar amendments. **a** Vegetative, **b** Flowering, **c** Harvesting stage. β-carotene and salicylic acid were applied as foliar spray while *D. salina* was added to the soil. Data are mean ± SE. Data labeled with different letters are significantly different at *P* value ≤ 0.05

In contrast to the flowering stage which contained higher *Chl a* and *b* content, the vegetative stage of *C. pepo* exhibited significantly higher carotenoid content than the flowering stage, followed by the harvesting stage in amended and non-amended plants (*p* < 0.001). Carotenoid content of *C. pepo* in the three stages of the growth was gradually decreased with increasing salinity from 0.55 to 33.5 dS m^−1^ in the non-amended plants whereas it was significantly increased with increasing salinity in the amended plants, particularly at 2.5 dS m^−1^ (Fig. 5). The combination of amendments increased the carotenoid content in the vegetative, flowering, and harvesting growth stages by 1.6, 2, and 3.7 folds versus 1.8, 2.4, and 5.8 folds for SA at 2.5 dS m^−1^, respectively, compared to the non-amended plants (Fig. 5a–c).

### Effect of salinity and amendments on nitrogen, phosphorus, and potassium content

**Fig. 6:**
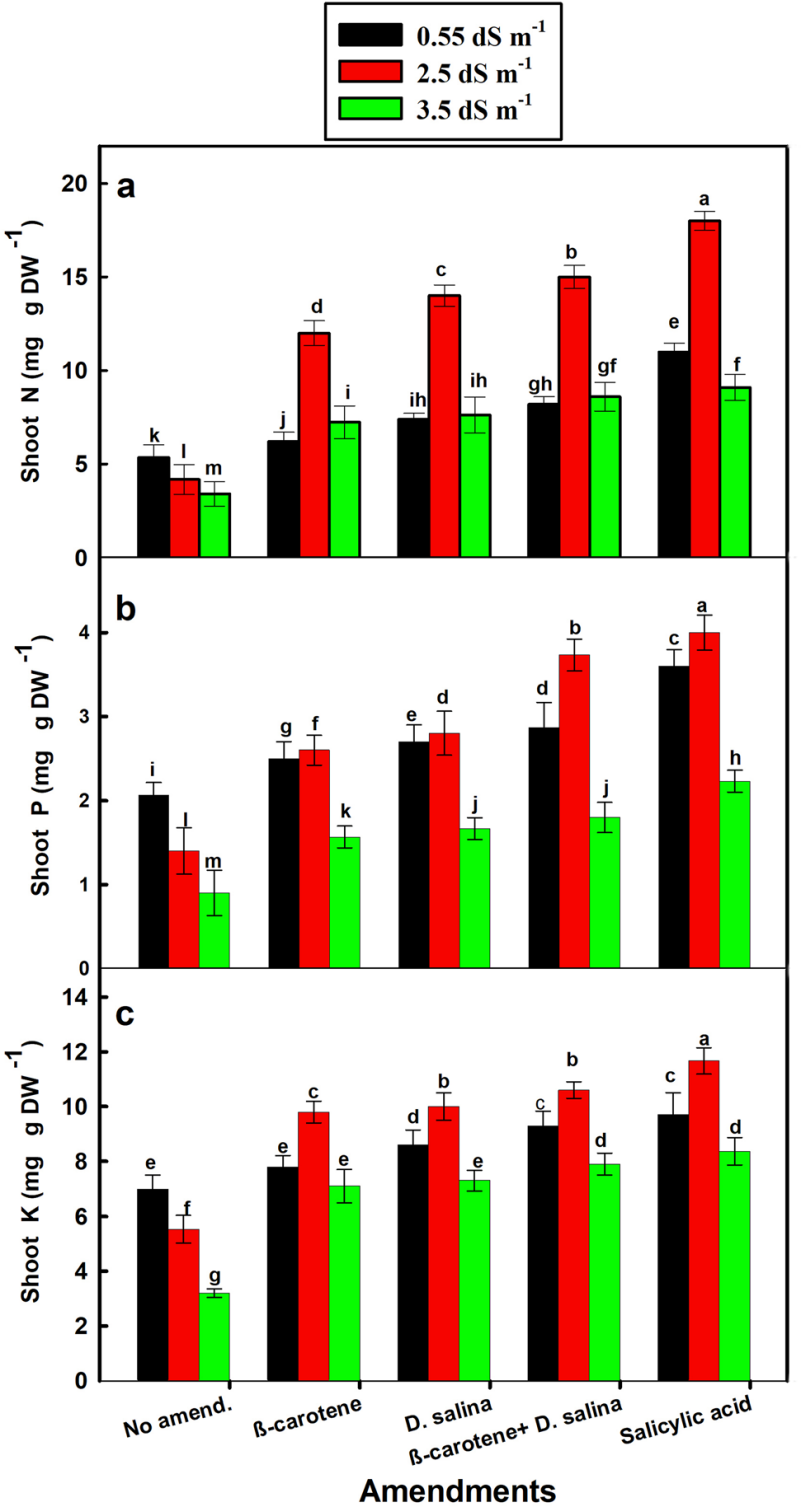
Nitrogen, phosphorus, and potassium content in *C. pepo* (L.) irrigated with graded dilutions of seawater and with different foliar amendments. β-carotene and salicylic acid were applied as foliar spray while *D. salina* culture was added to the soil. Data are mean ± SE. Data labeled with different letters are significantly different at *P* value ≤ 0.05

Although increasing salinity from 0.55 to 3.5 dS m^−1^ led to a progressive reduction in N, P, and K content of *C. pepo* shoot in the non-amended plants, salinity increased N, P, and K content in the amended plants, particularly at 2.5 dS m^−1^ (*P* value < 0.001). Among the amendment regimes, the combination of *D. salina* culture and its β-carotene extract exhibited better beneficial effect than each one alone, where it significantly increased N, P, and K content by 2.6, 1.7 folds, and 90% versus 3.3, 1.9, 1.1 folds for SA, respectively, compared to the non-amended plants (control) (Fig. 6a–c).

### Change of phenolic compounds content in response to salinity and amendments

**Fig. 7:**
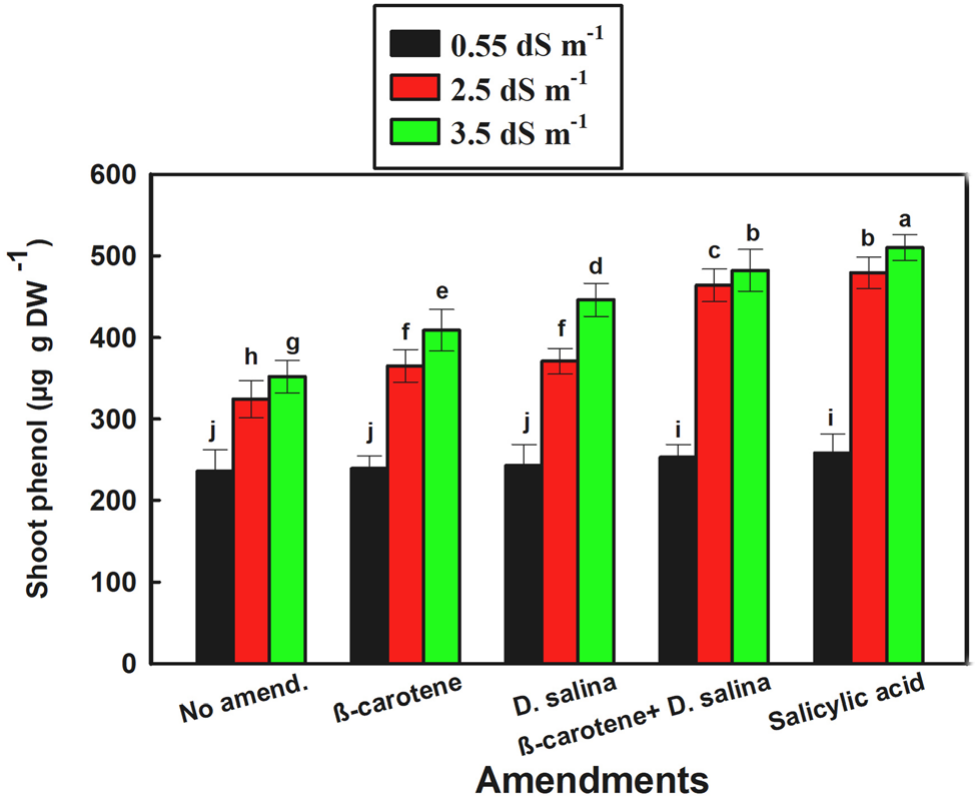
Phenol content in *C. pepo* (L.) irrigated with graded dilutions of seawater and amended with different foliar amendments. β-carotene and salicylic acid were applied as foliar spray while *D. salina* culture was added to the soil. Data are mean ± SE. Data labeled with different letters are significantly different at *P* value ≤ 0.05

The phenolics content of *C. pepo* exhibited significant progressive increases with increasing salinity from 2.5 to 3.5 dS m^−1^ in either non-amended or amended plants compared to the freshwater-irrigated plants either amended or non-amended plants (*P* value < 0.001). Within the different amendments, the combination of *D. salina* culture and its β-carotene extract significantly increased phenolics by 43% versus 48% folds for SA compared to control (Fig. 7). It was worth mentioning that the phenolics content of *C. pepo* didn't change significantly in the amended and non-amended plants that were irrigated with fresh irrigation water (0.55 dS m^−1^).

## Discussion

The beneficial effect of *D. salina* culture combined with its β-carotene extract in improving the growth performance (total biomass and fruit productivity) and physiological attributes (photosynthetic pigments, phenolics, N, P, and K^+^ contents) of the salt-stressed *C. pepo* was particularly evident under moderate salinity (2.5 dS m^−1^), with a magnitude approaching that of SA and exceeding that of either *D. salina* culture or its β-carotene extract alone; therefore it was focused on the effect of this combination at the moderate salinity in the discussion.

### Growth of salt-stressed *C. pepo* in response to the amendments

The significant reduction in growth in terms of total biomass or fruit biomass (FW and DW) of untreated *C. pepo* plants indicated that untreated *C. pepo* was adversely affected by salinity cumulating as a consequence of continuous irrigation with graded seawater. Also, the reduction in growth associated with the decrease in the photosynthetic pigments (*Chl a*, *Chl b*, and carotenoids), N, P, and K^+^ contents suggests the inability of *C. pepo* to cope with salt stress since it is considered a salt-sensitive species. This was in agreement with the findings of Taïbi et al. (2016) who found that salinity induced a marked reduction in dry matter and productivity along with oxidative stress of *Phaseolus vulgaris*. Salinity inhibits plant growth, productivity, photosynthesis, and chlorophyll biosynthesis (Desoky et al. 2018). The increased salinity decreased the photosynthetic pigments, in turn, photosynthesis that can be gauged by photosynthetic pigments (Qados 2011).

On the contrary, the significant increase in total biomass and fruit biomass (fresh or dry) of *C. pepo* treated with the combination of *D. salina* culture and its β-carotene extract could be due to the ability of treated *C. pepo* cells to increase the size of its sap vacuoles, which allows for collecting water, and this, in turn, dissolves salt ions that have accumulated and lead to the increase in fresh weight, in turn, dry matter (El-Adl et al. 2021). Thus, the results suggest that the high biomass of *C. pepo* treated with the combination at the moderate salinity could be attributed to high dry matter, not to high water content (Fig. S1). This is supported by high water content in treated *C. pepo* that was coincided with high salinity and low biomass (Figs. 1, 2, S1). El-Adl et al. (2021) found that high water content in *Ulva lactuca* exposed to high salinity (600 mM NaCl) was associated with low dry weight compared to those exposed to low salinity (300 mM NaCl).

The higher value of the harvest index in the salt-stressed *C. pepo* treated with the combination suggests that the combination of *D. salina* culture and its β-carotene extract could provide tolerance to plants just like the phytohormones such as SA. This could be attributed to that the higher value of harvest index was associated with the higher value of dried biomass of the plant and fruits in the salt-stressed *C. pepo* treated with the combination, reaching up to 90%–95% of the SA-treated plants at the moderate salinity (Fig. S2).

Salicylic acid regulates pant growth and enhances yield and yield attributes of crops when applied exogenously (Panda et al. 2012). Salicylic acid, as a hormone-like plant growth regulator, could alleviate the salt stress effect and induce *Cucumis sativus* growth (Canakci and Munzuroglu 2007). Also, SA acts as an endogenous signal molecule responsible for inducing stress tolerance in plants (Shaki et al. 2018). Moreover, it increased the foliage fresh and dry weight, fruit number, average fruit weight, fruit yield, vitamin C, carotenoid content, cuticle thickness of fruit pericarp, and translocation of sugars from leaves to fruits. Salicylic acid also caused a reduction in peroxidase and increasing in the invertase activities of pepper leaves and fruits (Elwan and El-Hamahmy 2009).

Although the combination could mitigate the negative effects of salt stress, improving the growth performance and productivity of *C. pepo* at intermediate salinity, it provides limited protection at high salinity levels, which could be attributed to the salt sensitivity of *C. pepo*.

### Performance of salt-stressed *C. pepo* in response to the amendments

The low levels of photosynthetic pigments (*Chl a*, *Chl b*, and carotenoids) during the growth stages (vegetative, flowering, and harvesting) of the non-treated *C. pepo* plants that were irrigated with the graded seawater compared to freshwater-irrigated plants could be an indicator for low photosynthesis, which inhibited plant growth. The adverse effect of salinity on plants could lead to an increase in the chlorophyllase activity on chlorophylls, causing chlorophyll degradation (Bulgari et al. 2019). Salinity inhibits chlorophyll biosynthesis, in turn, inhibiting plant photosynthesis (Desoky et al. 2018).

On the contrary, the significant increase in the *Chl a*, *Chl b*, and carotenoid contents during the growth stages of treated *C. pepo* plants that were irrigated with the diluted seawater indicates that the combination could increase the photosynthetic pigments, in turn, photosynthesis, thereby enhancing *C. pepo* growth. This could mean that the combination may inhibit the degradation of chlorophyll resulting from salt stress, increasing the content of the photosynthetic pigments, thereby photosynthetic machinery, photosynthesis, and growth (Whapham et al. 1993**)**.

The combination of *D. salina* culture and its β-carotene extract increased *Chl a* content by 84, 99, and 90% versus 96, 96, and 90% for *Chl b* and 91, 89, and 70% for carotenoids during the growth stages (the vegetative, flowering, and harvesting), respectively, of the SA-treated plants at 2.5 dS m^−l^. In comparison, plants with salinity tolerance have internal mechanisms allowing them to maintain photosynthesis in the presence of high levels of salt (Lee and Shen 2004), but the sensitive *C. pepo* could need exogenous amendments to cope with salt stress.

The coincidence of higher content of carotenoids and the lower content of *Chl a* and *Chl b* in the vegetative stage, but the reverse was true in the flowering stage, could be attributed to that the treated young plants of *C. pepo* could synthesize or evoke several metabolites such as antioxidants (carotenoids), particularly when the plant exposed to salt stress at the beginning of growth, suggesting the combination could enhance the adaptation of plant. In this context, Salem et al. (2014) attributed the higher carotenoid content to the effect of salinity stress compared to the control plants, since the non-enzymatic antioxidants, carotenoids, play an essential role in the plant defense system against oxidative stress (Mane et al. 2011).

*Cucurbita pepo* could direct all the physiological attributes into flowering and then fruiting stages, decreasing the synthesis of carotenoids but increasing the synthesis of *Chl a* and *Chl b*, in turn, increasing the photosynthetic rate and hence increasing the vegetative growth, thereby increasing fruiting in the flowering stage.

The results indicated that the variation in the *Chl a* and *Chl b* contents during the different growth stages led to the change in the ratio of *Chl a*/*Chl b* (Fig. S3), suggesting that the salinity affects the physiological and biochemical characteristics of *C. pepo* but the response was varied at different stages of growth. Salt stress affected *Chl a* more than *Chl b* as was reported by Jamil et al. (2012). Moreover, plants could employ biochemical, physiological, and molecular mechanisms to cope with salt stress, such as induction of anti-oxidative enzymes and synthesis of compatible solutes (Lee and Shen 2004).

The significant decrease in N, P, and K^+^ contents of non-amended *C. pepo* that was irrigated with diluted seawater compared to those irrigated with freshwater could be attributed to the negative effect of the increased salinity, cumulating from the continuous irrigation by diluted seawater. The increased salinity caused K^+^ deficiency due to the antagonistic effect of Na^+^ and K^+^ ions (Romero et al. 1997), causing inhibition of K^+^ uptake, decreasing photosynthetic activity, and consequently inhibiting the plant growth (Ueda et al. 2003).

Also, increasing salinity resulted in decreasing N concentrations. This could be due to N and Cl^−^ antagonism (Fisara et al. 2005). Under salt stress conditions, the decrease of nutrient uptake resulted in decreased transport rate of nutrients to the plant and, therefore, less growth. Moreover, the reduction of N availability significantly decreased plant photosynthesis, total leaf area, and consequently plant growth (Fisara et al. 2005).

On the contrary, the significant increase in N, P, and K^+^ contents of treated *C. pepo* that was irrigated with the diluted seawater compared to those untreated plants could be attributed to that the combination could facilitate ion homeostasis in salt-stressed *C. pepo* plants by increasing the osmotic potential in the cell, entering more water to the cell and consequently active absorption of nutrients including K^+^ which is important ion for the maintenance of osmotic equilibrium (Kusvuran 2010).

The results indicated that the combination had a profound ability to increase N, P, and K^+^ availability in *C. pepo*, reaching up to 83, 93, and 90% of the SA-treated plants, respectively, at the moderate salinity (2.5 dS m^−1^), thereby increasing photosynthetic rate and hence the plant growth and productivity. Under salt stress, the increased nutrient uptake resulted in increasing the transport rate of nutrients to the plant, thereby increasing plant growth and productivity (Fisara et al. 2005). Furthermore, high biomass at the intermediate salt level could be ascribed to the increased N/P ratio (Fig. S4), since the increased N availability increased the N/P ratio consequently enhancing the plant growth (Hopkins 1995).

### Effect of amendments on the phenolics content of salt-stressed *C. pepo*

**Fig. 8:**
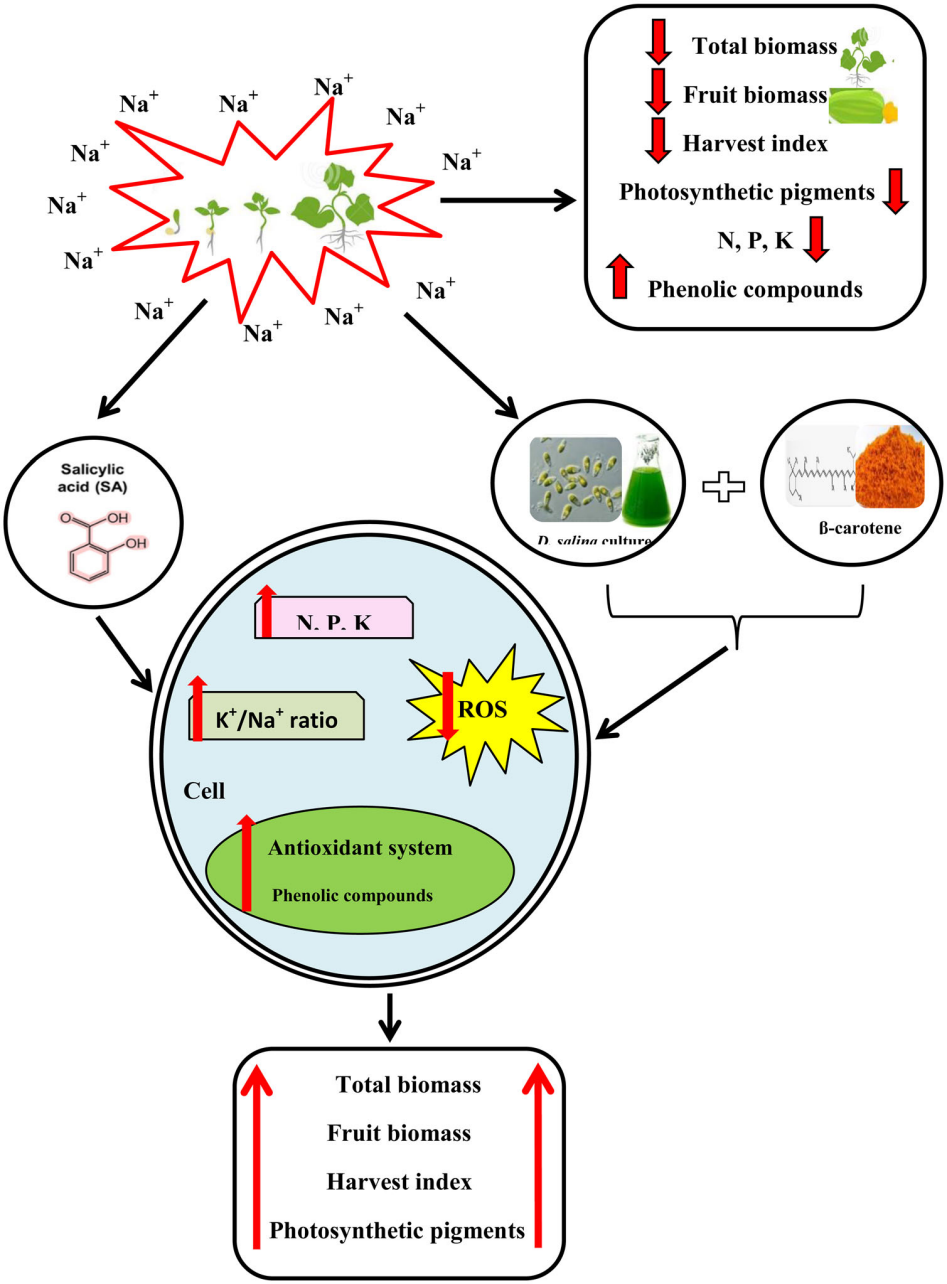
The mechanistic diagram shows the role of combined effect of *D. salina* culture and its β-carotene extract in enhancing the antioxidant activity of salt-stressed *C. pepo* (L.) compared to SA

The significant increase in phenolic compounds content in amended and salt-stressed *C. pepo* plants compared to those in the salt-stressed and non-amended plants could be attributed to the effectiveness of the combination in enhancing the antioxidant defense system in plants. Furthermore, the stability of phenolic compounds content in non-salt-stressed plants (irrigated with freshwater) either amended or not, signify that salt stress could induce the production of phenolic compounds; also it confirms that the combination could improve the salt tolerance efficiency of *C. pepo* by improving antioxidant defense systems via increasing its phenolic compounds compared to the untreated plants (Fig. 8).

This is in agreement with the findings of Salem et al. (2014) who confirmed that the increasing salinity increased total phenolic compounds. Phenolic compounds constitute a part of cellular solutes and help to reduce environmental stress (Sonar et al. 2011). The phenolic compounds are included in the non-enzymatic antioxidant system (Talbi et al. 2015). For scavenging high ROS levels resulting from salt stress, an efficient antioxidant system is required (Jan et al. 2017).

Accordingly, this was exemplified by the plants treated with the combination of *D. salina* culture and its β-carotene extract that increased the phenolics content by 97% of the SA-treated plants at the moderate salinity (2.5 dS m^−1^). This could signify that the combination of the *D. salina* culture and its β-carotene extract could have a profound ability to increase the antioxidant defense system in *C. pepo* similar to the hormone-like plant growth regulator, "Salicylic acid" (Dutta et al. 2015). Also, the benefit of *D. salina* and its β-carotene extract to salt-stressed *C. pepo* could be related to macro-and micro-nutrients, growth hormones, and phenolic compounds (El-Katony et al. 2020).

The beneficial effect of the combination of *D. salina* culture and its β-carotene extract in the alleviation of salt stress on the growth performance and physiological attributes of the salt-stressed *C. pepo* was more pronounced under moderate salinity (2.5 dS m^−1^) than either lower (0.55 dS m^−1^) or higher (3.5 dS m^−1^) salinity. It seems that *C. pepo* could exploit this combination for salt tolerance, enhancing the adaptation to the moderate salinity through the restoration of ion and osmotic homeostasis and modulation of plant metabolism including accumulation of osmoprotectants and activation of gene networks involved in the biosynthesis of ABA, Ca^2+^ signaling elements, and Na^+^ and K^+^ membrane transporters reduced the accumulation of toxic Na^+^ in shoots and leaves (Cebrián et al. 2021).

It was observed from the previous findings that there was no big difference between the effect of the combination of *D. salina* culture and its β-carotene extract on salt-stressed *C. pepo* and the effect of salicylic acid. Also, it was observed that the effectiveness of fresh β-carotene compared to dried β-carotene in ameliorating the deleterious effects of salt stress in *C. pepo* plants; this could be attributed to the drying process which reduces the antioxidant activity of β-carotene via oxidation. β-carotene could be degraded by a free radical oxidation mechanism and the degree of oxidation could depend on the heating time, heating temperature, and oxygen content (Suvarnakuta et al. 2005). El-Arroussi et al. (2018) used *D. salina* exo-polysaccharides to enhance *Solanum lycopersicum* tolerance to salt stress and they attributed the enhancement of the salinity tolerance to the decrease in length and dry weight of the plant’s shoot and root systems, increase in the concentration of K^+^, K^+^/Na^+^ ratio, phenolic compounds, and antioxidant activities. They also concluded the possibility of the use of these compounds as plant growth bio-stimulators under harsh environmental conditions.

Carotenoids, including β-carotene, are important constituents of photosynthetic organelles and play a major role in the protection of plants against photo-oxidative processes. They are efficient physical and chemical quenchers (antioxidants), scavenging singlet molecular oxygen and peroxyl radicals (Paiva and Russell 1999).

Also, they are potent scavengers of other reactive oxygen species (ROS) (Cvetkovic et al. 2013). β*-*carotene exhibits many biological activities for the plant, including quenching free radicals under salt stress conditions and preventing oxidative damage to the plant cell, owing to its vigorous antioxidant properties (Rammuni et al. 2019). The study confirmed that salt stress induces phenolic compounds, improving the salt tolerance in *C. pepo*.

This could be attributed to the positive influence of phenolic compounds on biochemical and physiological processes including, membrane permeability, ion uptake, enzyme activities, photosynthesis, growth, flowering, and development of plants (Elwan and El-Shatoury 2012).

The results revealed the potential similarity between the effects of SA or the combination of *D. salina* culture and its β–carotene extract, where both of the treatments increased the plant growth and yield via increasing the phenolic compounds, carotenoids (i.e. defensive system), N, P, and K^+^ contents (Fig. 8). Therefore, the study could suggest using the combination of the natural blooms of *D. salina* and its β-carotene extract (that is naturally secreted in situ, fresh) in improving the salt tolerance of *C. pepo*. It would be more economically beneficial and cost-effective in improving the salt tolerance of salt-sensitive *C. pepo* plants than the expensive exogenous chemical hormones.

## Conclusion

The results conclude that the severity of salts in the seawater used for crop irrigation could be efficiently alleviated by some supplements such as the combination of the culture of *D. salina*, and its β-carotene extract. This combination was better than the culture of *D. salina* or β-carotene extract alone in ameliorating the deleterious effect of salt stress in *C. pepo* plants. At the moderate salt level, the effects of the combination was beneficially similar to the SA effect, enhancing salt tolerance, adaptation, growth, and crop productivity. The salt-stressed *C. pepo* plants that received the combination showed an improvement in their salt tolerance via enhancing carotenoids and phenolic compounds production, resulting in an enhancement in their defensive system as well as N, P, and K^+^ contents. This study was focused on the growth and physiological attributes resulting from applications of algal and their extracts in alleviating negative effects of salt stress on *C. pepo* compared to SA-applications. However, this study needs further investigation to evaluate the effects of these three combinations i.e. the combination of the culture of *D. salina* and its β-carotene extract, the culture of *D. salina*, β-carotene, and non-amended plants on the regulation of the salt-inducible genes in *C. pepo*.

This study could serve as a starting point for developing crop cultivation, as well as remediating and resolving problems associated with the severity of salt stress on crop plants, including the salt-sensitive *C. pepo*, and crop production for increasing global food security. The application of algae and/or their extracts on *C. pepo* may be effective cost and give high productivity that may be close to SA-application.

Finally, this study recommends cultivating *D. salina* on large scale in natural or synthetic open systems, exploiting natural sources such as sunlight until the β-carotene was secreted in *D. salina* culture, then using the *D. salina* culture with its β-carotene extract as amendments in improving the growth and productivity of the salt-stressed *C. pepo*, reducing the cost of *D. salina* culturing in the laboratory.

## Supplementary Information

Below is the link to the electronic supplementary material.

Supplementary file1 (DOCX 1298 kb)

## Data Availability

Data will be made available on reasonable request.
