# Supplementary material for: Impact of the microalga Dunaliella salina (Dunal) Teodoresco culture and its β-carotene extract on the development of salt-stressed squash (Cucurbita pepo L. cv. Mabrouka)
Source: Physiol Mol Biol Plants. 2022 Apr 20;28(4):749–62. doi: 10.1007/s12298-022-01176-6 (PMC9110587; doi:10.1007/s12298-022-01176-6)
Supplement: Supplementary file 1 — Supplementary file1 (DOCX 1298 kb) [file 12298_2022_1176_MOESM1_ESM.docx]

**Impact of the microalga *Dunaliella salina (*Dunal) Teodoresco culture and its β-carotene extract on the development of salt-stressed squash (*Cucurbita pepo* L. cv. Mabrouka)**

**Table S1** Significance of the effect of salinity and amendments, salinity × amendment and their interactions on *C. pepo* growth performance and development by two-way ANOVA at *P* < 0.05

| **Source of Variation** | **df** | ***F*** | ***P*** |  | ***F*** | ***P*** |  | ***F*** | ***P*** |
| --- | --- | --- | --- | --- | --- | --- | --- | --- | --- |
|  |  | ***Chl a* vegetative** | |  | ***Chl a* flowering** | |  | ***Chl a* harvesting** | |
| **Salinity** | 2 | 549.4 | 0.000 |  | 1169.0 | 0.000 |  | 116.7 | 0.000 |
| **Amendment** | 4 | 1939.5 | 0.000 |  | 3178.4 | 0.000 |  | 213.9 | 0.000 |
| **Salinity × Amendment** | 8 | 96.55 | 0.000 |  | 138.4 | 0.000 |  | 11.22 | 0.000 |
|  |  | ***Chl b* vegetative** | |  | ***Chl b* flowering** | |  | ***Chl b* harvesting** | |
| **Salinity** | 2 | 49.9 | 0.000 |  | 563.7 | 0.000 |  | 429.6 | 0.000 |
| **Amendment** | 4 | 429.3 | 0.000 |  | 803.4 | 0.000 |  | 537.5 | 0.000 |
| **Salinity × Amendment** | 8 | 4.95 | 0.000 |  | 21.8 | 0.000  4 |  | 19.8 | 0.000  4 |
|  |  | ***Chl a/b* vegetative vegetative** | |  | ***Chl a/b* flowering** | |  | ***Chl a/b* harvesting** | |
| **Salinity** | 2 | 165.54 | 0.000 |  | 79.08 | 0.000 |  | 275.45 | 0.000 |
| **Amendment** | 4 | 244.99 | 0.000 |  | 45.76 | 0.000 |  | 445.47 | 0.000 |
| **Salinity × Amendment** | 8 | 51.30 | 0.000 |  | 16.04 | 0.000 |  | 45.36 | 0.000 |
|  |  | **β- Carotene vegetative** | |  | **β- Carotene flowering** | |  | **β- Carotene harvesting** | |
| **Salinity** | 2 | 4552.7 | 0.000 |  | 42.6 | 0.000 |  | 27.5 | 0.000 |
| **Amendment** | 4 | 1476.4 | 0.000 |  | 148.3 | 0.000 |  | 526.0 | 0.000 |
| **Salinity × Amendment** | 8 | 168.6 | 0.000 |  | 3.6 | 0.000 |  | 8.7 | 0.000 |
|  |  | **Nitrogen (N)** | |  | **Phosphorus (P)** | |  | **N/P** | |
| **Salinity** | 2 | 650.2 | 0.000 |  | 1082.4 | 0.000 |  | 335.96 | 0.000 |
| **Amendment** | 4 | 415.6 | 0.000 |  | 624.4 | 0.000 |  | 33.70 | 0.000 |
| **Salinity × Amendment** | 8 | 54.7 | 0.000 |  | 50.8 | 0.000 |  | 16.29 | 0.000 |
|  |  | **Potassium (K)** | |  | **WC%** | |  | **Harvest index** | |
| **Salinity** | 2 | 122.2 | 0.000 |  | 3177.2 | 0.000 |  | 2554.2 | 0.000 |
| **Amendment** | 4 | 123.7 | 0.000 |  | 818.38 | 0.000 |  | 232.45 | 0.000 |
| **Salinity × Amendment** | 8 | 7.7 | 0.000 |  | 1048.38 | 0.000 |  | 125.3 | 0.000 |
|  |  | **FW fruits** | |  | **DW fruits** | |  | **Phenol content** | |
| **Salinity** | 2 | 7699.3 | 0.000 |  | 22401.2 | 0.000 |  | 7592.8 | 0.000 |
| **Amendment** | 4 | 3495.4 | 0.000 |  | 8735.7 | 0.000 |  | 899.1 | 0.000 |
| **Salinity × Amendment** | 8 | 145.8 | 0.000 |  | 1030.6 | 0.000 |  | 160.2 | 0.000 |
|  |  | **FW biomass** | |  | **DW biomass** | |  |  | |
| **Salinity** | 2 | 108474 | 0.000 |  | 1956.3 | 0.000 |  |  |  |
| **Amendment** | 4 | 151714 | 0.000 |  | 2050.9 | 0.000 |  |  |  |
| **Salinity × Amendment** | 8 | 3208 | 0.000 |  | 63.4 | 0.000 |  |  |  |

Number of replicates (*n*) was from 3 to each analysis

*F* Fisher’s criterion, *P* probability at < 0.05

**
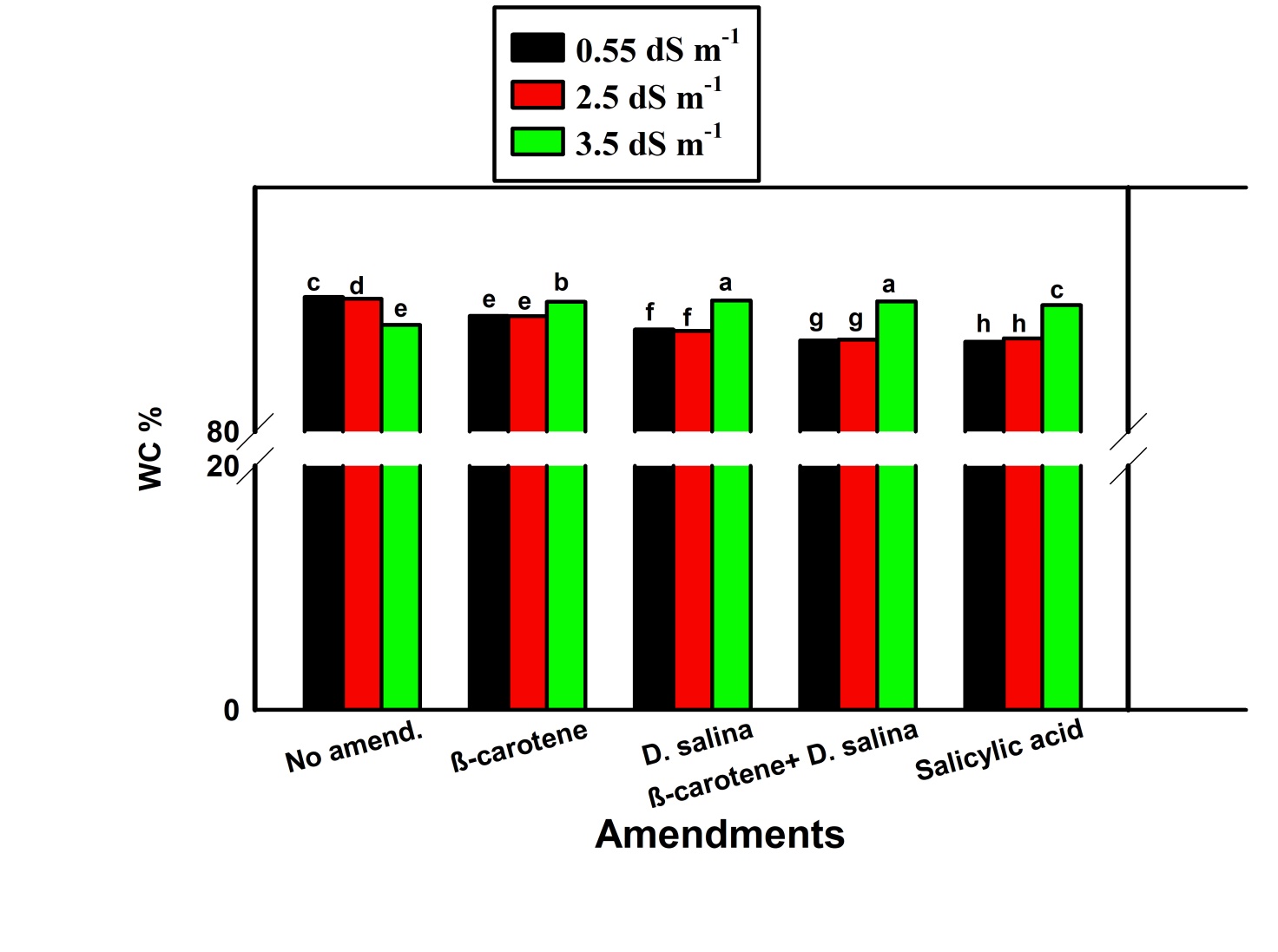
**

**Fig. S1** Water content (WC%) of *C. pepo* (L.) grown on clay soil, irrigated with graded dilutions of seawater and amended with different foliar amendments. β-carotene and salicylic acid were applied as foliar spray while *D. salina* was added to the soil. Data are mean ± SE. Data labeled with different letters are significantly different at *p* ≤ 0.05.

**
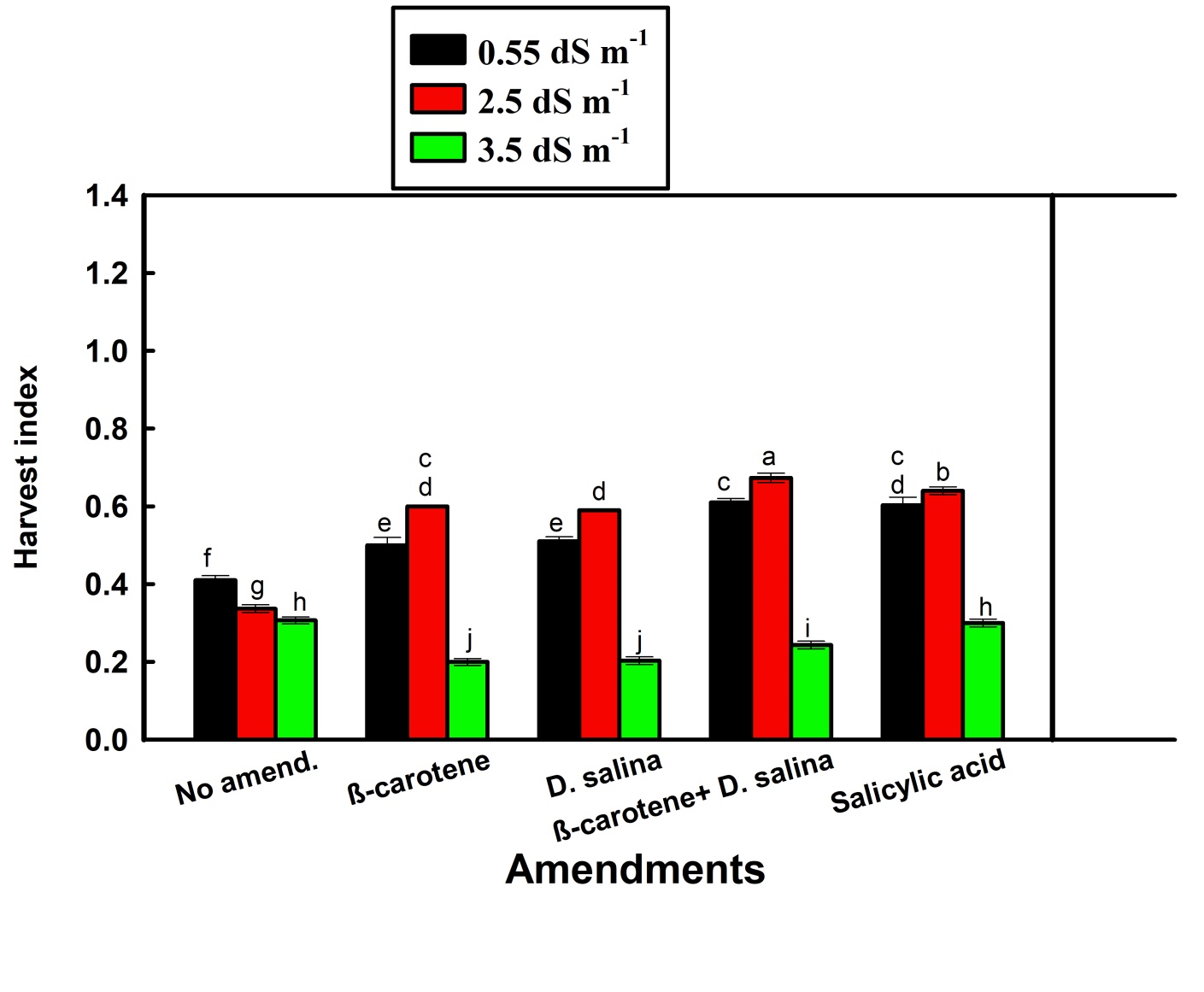
**

**Fig. S2** Harvest index of *C. pepo* (L.) grown on clay soil, irrigated with graded dilutions of seawater and amended with different foliar amendments. β-carotene and salicylic acid were applied as foliar spray while *D. salina* was added to the soil. Data are mean ± SE. Data labeled with different letters are significantly different at *p* ≤ 0.05.

**
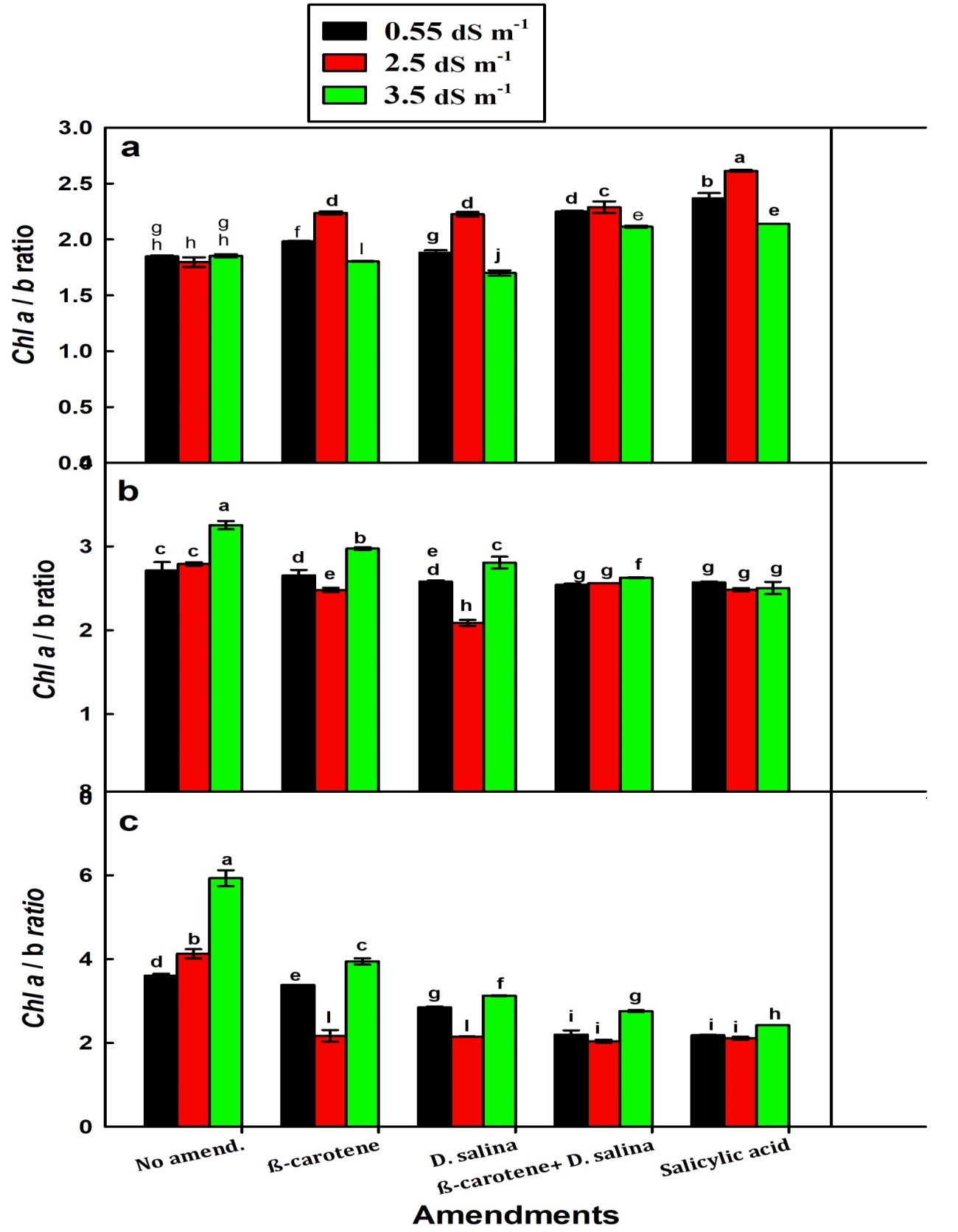
**

**Fig. S3** *Chl a/ b* ratio of *C. pepo* (L.) grown on clay soil, irrigated with graded dilutions of seawater and amended with different amendments. **a** Vegetative, **b** Flowering, **c** Harvesting stage. β-carotene and salicylic acid were applied as foliar spray while *D. salina* was added to the soil. Data are mean ± SE. Data labeled with different letters are significantly different at *p* ≤ 0.05.

**
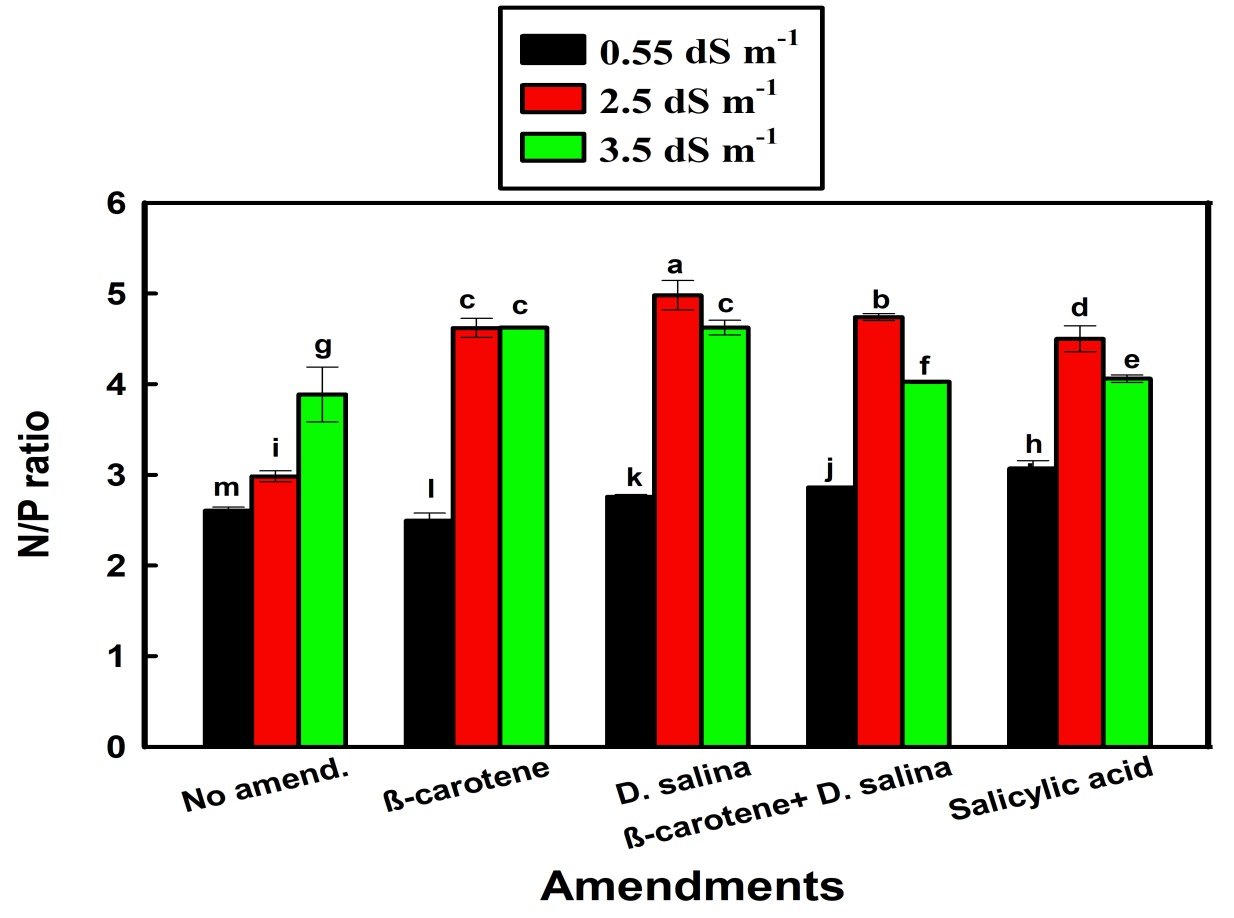
**

**Fig. S4** N/P ratio of *C. pepo* (L.) grown on clay soil, irrigated with graded dilutions of seawater and amended with different foliar amendments. β-carotene and salicylic acid were applied as foliar spray while *D. salina* was added to the soil. Data are mean ± SE. Data labeled with different letters are significantly different at *p* ≤ 0.05.

**Graphical Abstract**

**
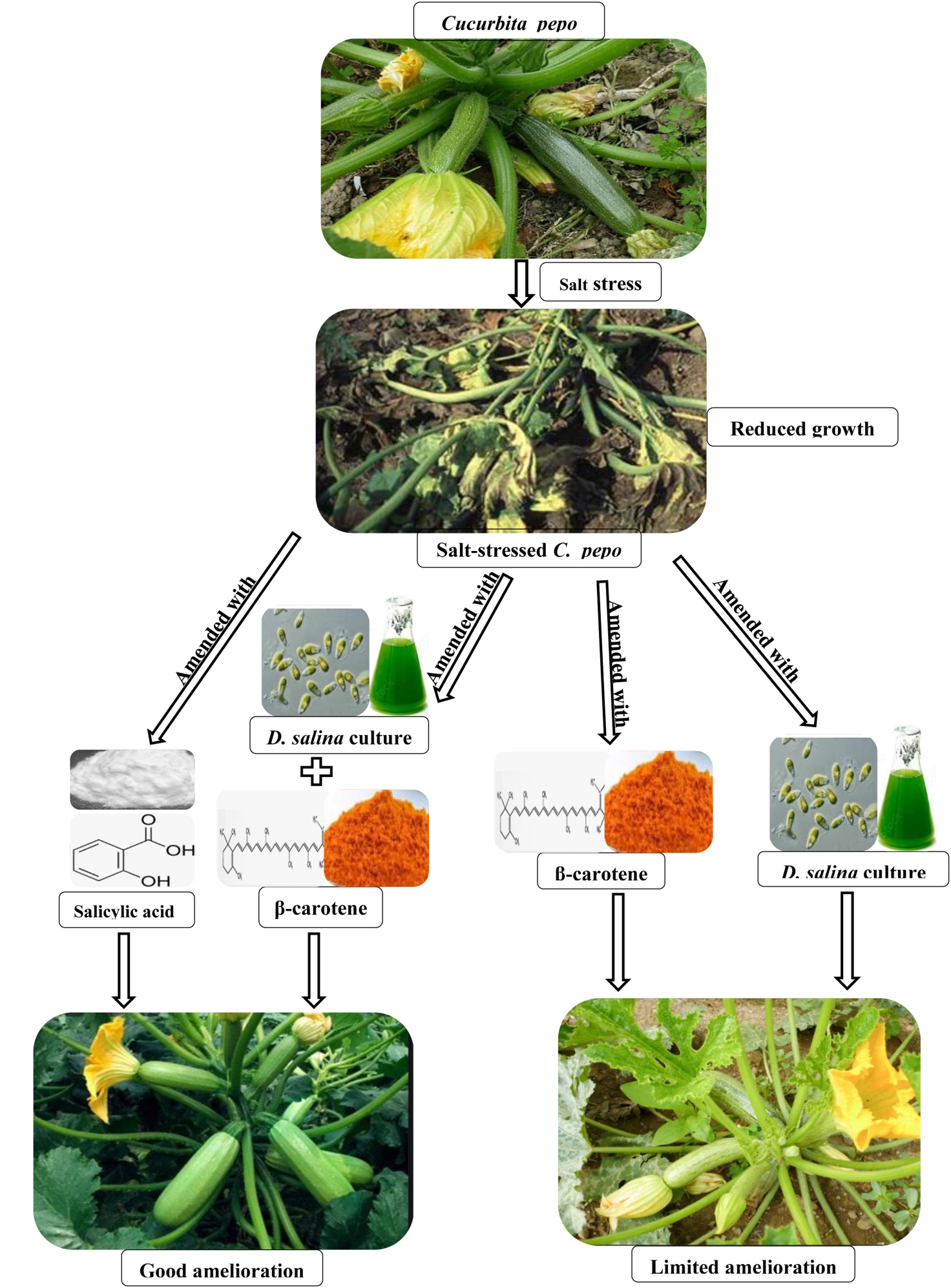
**
